# Nutritional Interventions for Pressure Ulcer Prevention in Hip Fracture Patients: A Systematic Review and Meta-Analysis of Controlled Trials

**DOI:** 10.3390/nu17040644

**Published:** 2025-02-11

**Authors:** Jose M. Moran, Laura Trigo-Navarro, Esther Diestre-Morcillo, Elena Pastor-Ramon, Luis M. Puerto-Parejo

**Affiliations:** 1 Nursing and Occupational Therapy College, University of Extremadura, 10001 Caceres, Spain; 2 Área de Salud de Badajoz, Supervisora del Bloque Quirúrgico, Hospital Materno Infantil de Badajoz, Calle Violeta 3, 06010 Badajoz, Spain; laura.trigo@salud-juntaex.es; 3 Área de Salud de Badajoz, Banco de Sangre, Hospital Universitario de Badajoz, Av. de Elvas, s/n, 06080 Badajoz, Spain; 4 Biblioteca Virtual de ciencias de la Salud de las Illes Balears (Bibliosalut), Ctra. De Valldemossa, 79, mòdul L+1, 07120 Palma, Spain; elena.pastor@bibliosalut.com; 5 Gerencia del Área de Salud de Badajoz, Supervisor del Área de Investigación, Proyectos y Gestión, Av. de Huelva, 8, 06005 Badajoz, Spain; luis.puerto@salud-juntaex.es

**Keywords:** hip fracture, pressure ulcers, oral nutritional supplement, pressure sores, meta-analysis, wound healing, nutritional intervention

## Abstract

Background/Objective: Pressure ulcers represent a significant complication in patients with reduced mobility, such as those recovering from hip fractures. In the present study, we aimed to comprehensively assess the impact of oral nutritional interventions on the development of pressure ulcers in hip fracture patients via a systematic review and meta-analysis of controlled studies evaluating the effectiveness of oral nutritional supplements compared with standard care. Methods: In accordance with PRISMA standards, this systematic review and meta-analysis of controlled studies evaluated the effectiveness of any type of oral nutritional supplements compared with standard care in hip fracture patients. The risk of bias was evaluated using the Cochrane ROB2 tool for randomized controlled trials and the ROBINS-1 tool for nonrandomized trials. Results: Fourteen studies (10 randomized controlled trials and 4 controlled trials) published since 1990 (*n* = 1648) were included. Oral nutritional supplementation was associated with a statistically significant decrease in the odds ratio of developing pressure ulcers in hip fracture patients (OR 0.54, 95% CI: 0.40–0.73, *p* < 0.001). Conclusions: The incidence and evolution of pressure ulcers can be improved by oral dietary supplementation in patients who have undergone hip fracture surgery. Accordingly, we propose that oral nutritional supplementation should be considered an essential component of comprehensive post-hip-fracture care.

## 1. Introduction

Ulceration is a common problem in the aging population in the Western world [1]. Data from 2020 revealed that the prevalence of ulcers between 2008 and 2018 was 12.8% globally, with rates of 14.5% in Europe, 13.6% in North America, 12.7% in South America, 3% in Asia, 12.6% in the Middle East, and 9% in Australia [2]. An ulcer or pressure ulcer refers to local damage to the skin and underlying soft tissues caused by pressure on the skin of different parts of the body; this pressure is often caused by a bed but can also be caused by a chair or other hard objects. This pressure on the tissues reduces the blood supply to the skin, causing thinning of the epithelium, contraction of subcutaneous adipose tissue, and loss of collagen elasticity [3]. In patients who have suffered a hip fracture, immobilization is prolonged, and the factors associated with this type of patient often significantly increase the risk of pressure ulceration [4], highlighting the vulnerability of this population. Additional factors that contribute to ulcer development in this population include advanced age, comorbidities, and, in many cases, nutritional deficiencies [5]. These findings highlight the need for vigilant preventive measures and comprehensive care strategies for ulcers in this population.

Intrinsic factors, including malnutrition and protein intake, are frequent predisposing risk factors for the development of ulcers, as well as for the severity and prognosis of ulcers [6,7,8,9,10]. The patient’s nutritional approach is therefore crucial for recovery after hip fracture, especially to reduce fracture-related complications such as pressure ulcers. Oral supplements are often used for patients who can eat naturally but who, owing to their condition, are unable to meet their nutritional needs through a regular meal pattern. These supplements are often characterized by different proteins, calories, and micronutrients and have been shown to play important roles in the ability of tissues and even the immune system to recover. For patients who cannot tolerate regular oral nutrition, usually due to the presence of comorbidities such as dysphagia or severe post-operative complications, an enteral nutrition regimen can deliver essential nutrients directly to the gastrointestinal tract through a tube [11]. In the latter case, specific nutritional preparations are often rich in protein and various immunomodulatory components that act to promote wound healing and reduce inflammation. Both oral nutritional supplements (ONSs) and tube feeding require individualized monitoring of the hip fracture patient to ensure control of problems such as adherence to ONSs and the appearance of potentially serious complications such as aspiration in tube feeding [11]. Therefore, the development of an adequate and dynamic feeding strategy for this type of patient is focused not only on facilitating physical recovery but also on improving quality of life, as we reduce risks and promote the patient’s comprehensive rehabilitation.

The grade of evidence and strength of recommendations are determined by evaluating various factors related to the quality of the available trials, including risk of bias, result consistency, precision, directness, potential publication bias, and the strength of the observed associations. In this context, performing a meta-analysis is a justified approach [12], as it provides a robust framework for clarifying the efficacy of available treatments. To comprehensively assess the impact of oral nutritional interventions on the development of pressure ulcers in hip fracture patients, we conducted a systematic review and meta-analysis of controlled studies evaluating the effectiveness of oral nutritional supplements compared with standard care.

## 2. Materials and Methods

The review was conducted following the indications of the Preferred Reporting Items for Systematic Reviews and Meta-Analyses (PRISMA) statement [13] (Supplementary Material).

### 2.1. Search Strategy

Three investigators (JMM, LMP-P, and EP-R) independently conducted an electronic literature search using EMBASE, PubMed Medline, OVID Medline, and Scopus databases. The selection was not limited by language, and publications up to July 2024 were retrieved. Disagreements were resolved by consensus with a fourth author (LT-N). A description of the strategy used in the identification of potentially relevant publications is provided in the Supplementary Material.

### 2.2. Study Selection

**Figure 1:**
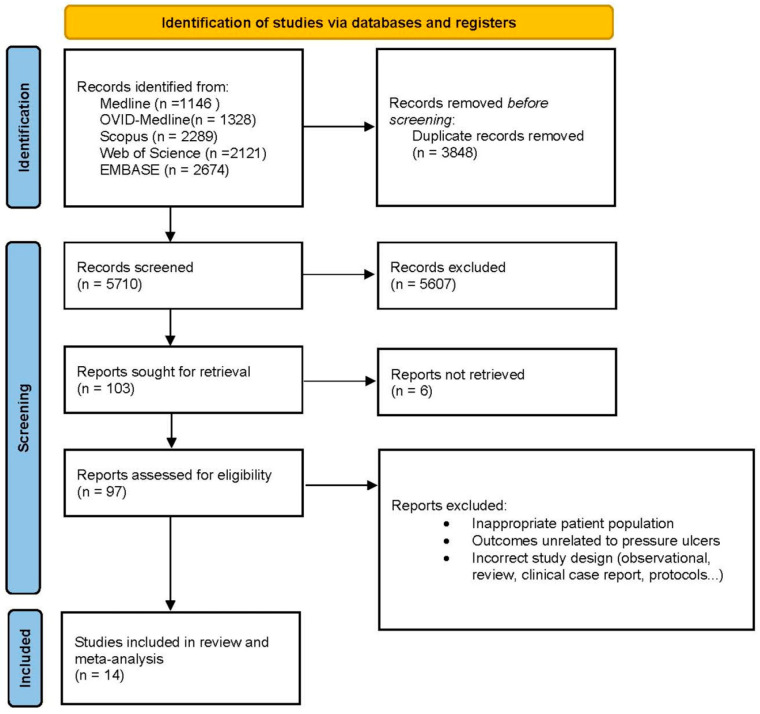
Flow diagram of the study selection process following the PRISMA guidelines, illustrating the identification, screening, eligibility, and inclusion of studies in the meta-analysis.

We included only controlled trials that (1) addressed the efficacy of an oral nutritional intervention compared with a control in patients after hip fracture surgery and (2) measured the development of pressure ulcers during the follow-up period. It was not necessary to contact the authors to ask for further information. The search results were uploaded to Rayyan software, an internet-based platform that enables collaboration between reviewers during the screening process [14]. Initially, duplicate articles were removed; in the subsequent step, JMM and LMP-P independently screened the titles and abstracts of all the records. Any conflicts that arose were resolved through consensus. When needed, a third author, LT-N, was consulted. Finally, the retrieved full texts were assessed for eligibility by both JMM and LMP-P. Studies that did not meet eligibility criteria were listed, as were the reasons for their exclusion. A PRISMA flow chart was developed to illustrate the entire selection process (Figure 1).

### 2.3. Data Extraction

Two authors (JMM and LMP-P) independently extracted data from the selected studies on a standardized record form. Disagreements were resolved by consensus with a third author (LT-N). The following information was extracted: (1) study population characteristics; (2) country; (3) clinical setting in which the study was performed; (4) duration and type of interventions; and (5) outcomes.

### 2.4. Outcomes

The primary and sole outcome was the absolute number of patients with pressure ulcers that developed during the study intervention period.

### 2.5. Risk-of-Bias Assessment

Bias was assessed according to the Cochrane Collaboration criteria [15], and the following dimensions were assessed in all studies included in the meta-analysis: randomization, allocation concealment, blinding of participants and staff, blinding of outcome assessment, incomplete outcome data, and selective reporting. Two assessors independently assessed the risk of bias as low risk, high risk, and unclear risk. Any discrepancies between the raters were resolved through consensus. No authors of the included articles were contacted to obtain additional information on unclear reporting. Overall, when a study did not include sufficient information to allow a decision to be made regarding the domains assessed, an unclear evaluation was assigned. Any discrepancies between the raters were resolved through consensus.

Nonrandomized controlled trials were assessed using the ROBINS-1 tool for assessing the risk of bias in nonrandomized studies [16].

Risk-of-bias assessment figures were created via the web app robvis (Risk-Of-Bias VISualization) [17]

### 2.6. Publication Bias

Publication bias was assessed using the trim-and-fill analysis [18] to adjust for funnel plot asymmetry and the fail-safe N method (Rosenthal approach) to estimate the number of null studies required to negate the observed effect.

### 2.7. Artificial Intelligence in Manuscript Refinement

Artificial intelligence tools (Grammarly and DeepL) were employed to refine the manuscript’s language, enhance clarity, and improve the communication of complex ideas.

### 2.8. Statistical Analysis

Odds ratios were calculated from the number of events. All estimates are provided along with 95% confidence intervals (95% CIs). For the present meta-analysis, a random effects model was used to pool all eligible studies. A Baujat plot was additionally used to detect studies that could be a potential source of heterogeneity in the meta-analysis [19]. To assess this heterogeneity, the I^2^ statistic was used to evaluate heterogeneity, with an I^2^ between 0% and 40% indicating nonsignificant heterogeneity; between 30% and 60%, indicating moderate heterogeneity; between 50% and 90% indicating significant heterogeneity; and between 75% and 100% indicating significant heterogeneity [20]. Sensitivity was evaluated by leave-one-out analysis. The Baujat plot, the sensitivity analysis forest plot, and the funnel plot for publication bias analysis were generated via the Pandas library, Matplotlib, and NumPy in Python.

## 3. Results

### 3.1. Study Descriptions

The initial search revealed studies from between 1990 and July 2024, a period spanning more than three decades. This initial search sets the totality of available records. After an initial review of titles and abstracts to exclude studies irrelevant to the research objective, 97 records were considered potentially relevant and underwent a detailed full-text search process. After this exhaustive review, a total of 14 studies (10 randomized controlled trials and 4 controlled trials) published since 1990 met the inclusion criteria and were included in the meta-analysis. The study selection process is summarized in Figure 1 according to a PRISMA flowchart describing all the steps of identification, screening, qualification, and inclusion according to commonly accepted standards for systematic reviews and meta-analyses.

Detailed information on the studies included in the systematic review and meta-analysis can be found in Table 1, which provides comprehensive information on key aspects of each study. Details on the type of intervention are included, as are the duration of the intervention and the follow-up period. Participant characteristics, such as the mean age and demographics, as well as country of origin, intervention characteristics, and study design, are also included. The outcome measures include primary outcomes such as pressure ulcer incidence and secondary outcomes such as changes in fat-free body weight, functional recovery, and nutritional status. Our meta-analysis included data from 14 published studies [5,21,22,23,24,25,26,27,28,29,30,31,32,33]. In one study, the data were analyzed as separate groups due to differences in the nature of the intervention (protein powder supplement (PPS) or energy protein supplement (EPS)) [21]. In this case, the unique control group was divided accordingly for the analyses.

### 3.2. Study Descriptions

The 14 trials included herein were undertaken in eight different countries, mostly Europe (*n* = 9) [5,21,24,25,26,27,28,29,30,31,32], Asia [23,33], and Oceania [22]. Altogether, the 14 trials involved 1648 hip fracture patients. The trials covered in the present review were conducted between 1990 and 2024. The follow-up period across the studies varied significantly, with a mean of approximately 82.7 days (14–210 days). However, in some studies, the follow-up period was extended only until hospital discharge, making it unclear how long these periods lasted for certain participants. The mean duration of the intervention was approximately 36.4 days (14–90 days). Additionally, in some studies, the duration was tied to hospitalization and ended at discharge, again making the exact intervention length also unclear for certain participants.

### 3.3. Nutritional Interventions and Pressure Ulcers in Hip Fracture Patients

The number of pressure ulcers diagnosed after nutritional interventions in hip fracture patients was pooled to obtain a total estimate of the overall effect of the intervention. A subgroup analysis was conducted in the meta-analysis, separating RCTs from controlled trials to minimize the risk of bias associated with study design differences.

**Figure 2:**
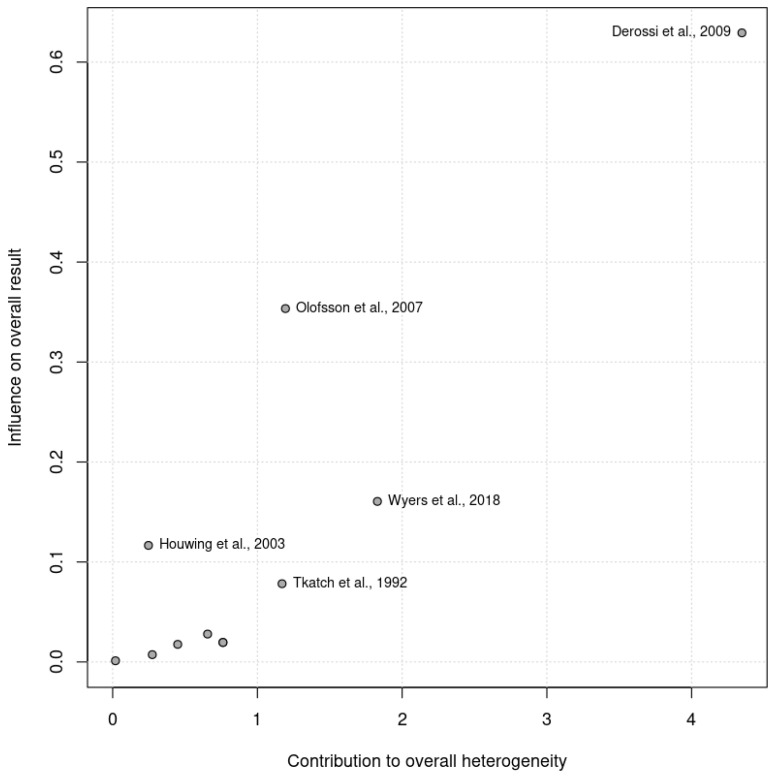
Baujat plot illustrating the contribution of individual studies to the overall heterogeneity (x axis) and their effect size (y axis). Studies further to the right contributed more significantly to heterogeneity.

The pooled analysis did not reveal significant heterogeneity (Chi^2^ = 13.70, df = 14, *p* = 0.47; I^2^ =0%). No heterogeneity was observed among the RCTs (Chi^2^ = 11.71, df = 10, *p* = 0.30; I^2^ =15%) or the controlled trials (Chi^2^ = 0.48, df = 3, *p* = 0.92; I^2^ =0%) in the subgroup analysis. Given that the I^2^ statistic indicated nonsignificant 15% heterogeneity, a Baujat plot (Figure 2) was generated to further explore this effect. The plot revealed that one study contributed more significantly to the observed heterogeneity, suggesting variability in their results when compared with the combined effect estimate.

**Figure 3:**
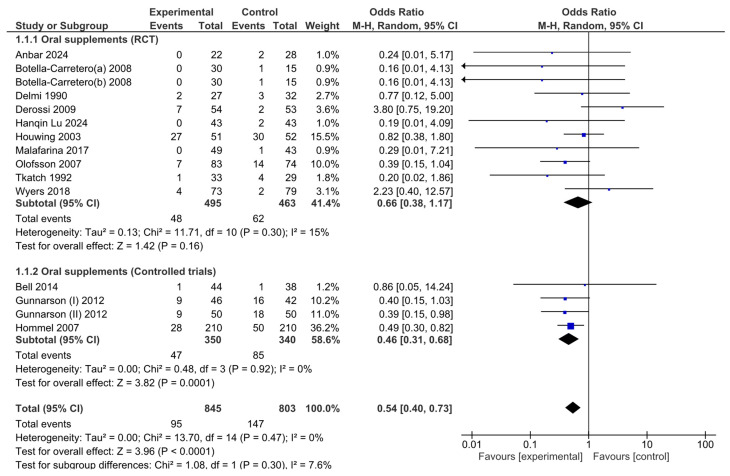
Forest plot of comparison between nutritional intervention and control in hip fracture patients. Odds ratios for pressure ulcer development from baseline to the end of the intervention.

An analysis of the pooled results via the random effects model revealed that there were significant effects associated with oral nutritional interventions and the development of pressure ulcers in hip fracture patients. The pooled OR (95% CI) was 0.54 (0.40, 0.73). The overall effect size for the OR calculated as Z was 3.96 (*p* ≤ 0.0001; Figure 3).

**Figure 4:**
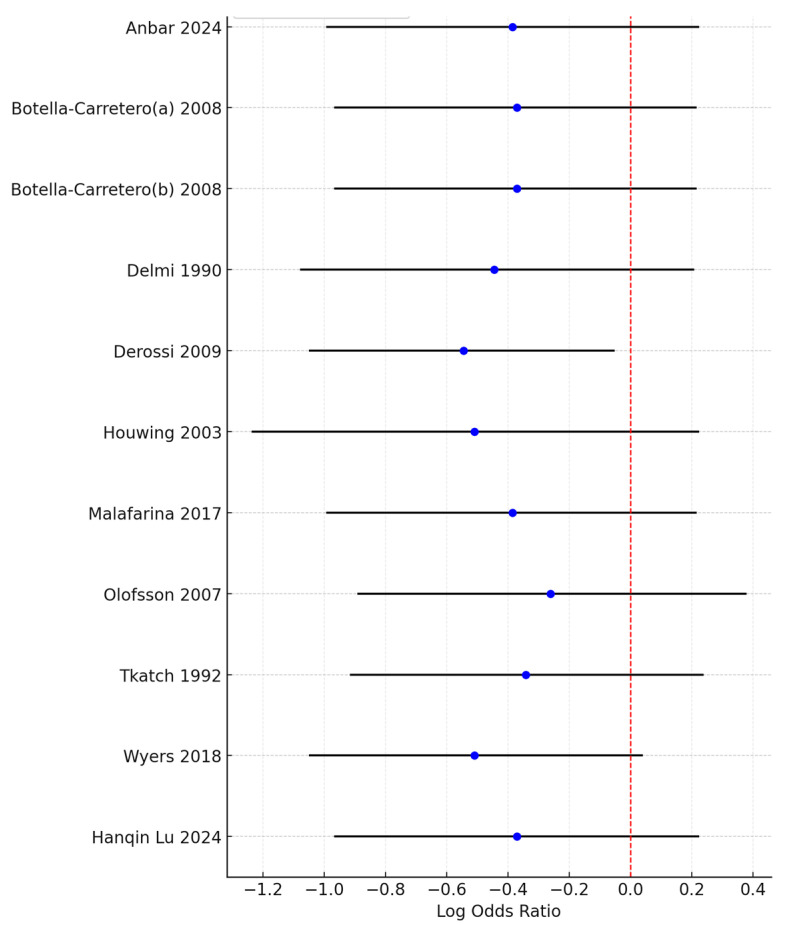
Forest plot from the leave-one-out sensitivity analysis showing the log odds ratio and 95% confidence intervals for each study when excluded from the meta-analysis. The red dashed line represents the null value (OR = 1), and the plot highlights the impact of individual studies on the overall results.

Although the combined meta-analysis of RCTs and controlled trials revealed a significant effect of the intervention on the risk of developing pressure ulcers, the subgroup analysis revealed that this effect was not observed in the RCT subgroup (OR = 0.66, 95% CI: 0.38–1.17)) but was present in the controlled trial subgroup (OR = 0.46, 95% CI: 0.31–0.68). To further investigate the effect on heterogeneity identified through the Baujat plot, a leave-one-out sensitivity analysis was conducted. The forest plot (Figure 4) shows that excluding the study by Derossi 2009 resulted in a globally statistically significant OR of 0.58, with a 95% CI of 0.35–0.95. Thus, this study had a considerable impact on the meta-analysis results and a significant effect on the overall outcome.

### 3.4. Category of Pressure Ulcers

**Figure 5:**
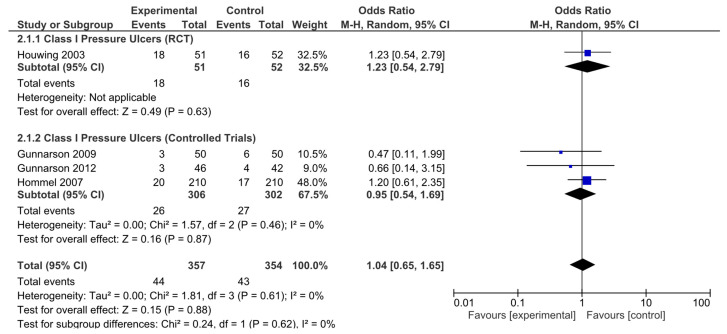
Forest plot for class I pressure ulcers showing the log odds ratio and 95% confidence intervals for each study.

In addition, analyses were performed according to the type of pressure ulcer. The results were subsequently analyzed in aggregate and segregated according to the study design. Among the studies analyzed, four (one randomized clinical trial and three nonrandomized controlled studies) were identified that provided specific data on pressure ulcer type, classifying pressure ulcers according to severity. No statistically significant differences in the risk of developing category I pressure ulcers were observed in the pooled analysis (OR = 1.04, 95% CI: 0.65–1.65). Neither were they observed for the RCTs (OR = 1.23, 95% CI: 0.54–2.79) or in the pooled analysis of nonrandomized studies (OR = 0.95, 95% CI: 0.54–1.69; Figure 5). No significant heterogeneity was reported in any of these analyses.

**Figure 6:**
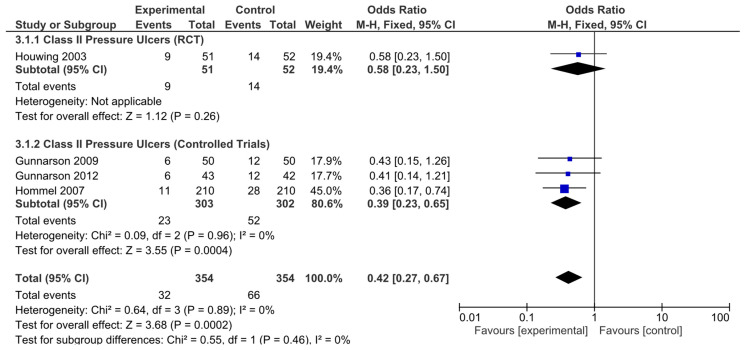
Forest plot for class II pressure ulcers showing the log odds ratio and 95% confidence intervals for each study.

The results obtained were different when the risk of developing hip fracture in patients with category II ulcers was analyzed. The results from the only RCT analyzed were not statistically significant (OR = 0.58, 95% CI: 0.23–1.50). However, the nonrandomized controlled trials did (OR = 0.39, 95% CI: 0.23–0.65). The pooled analysis also revealed a significant result, indicating a decreased risk of developing category II ulcers in these patients (OR = 0.42, 95% CI: 0.27–0.67; Figure 6). Again, no significant heterogeneity was revealed by any of the analyses performed.

### 3.5. Risk of Bias

**Figure 7:**
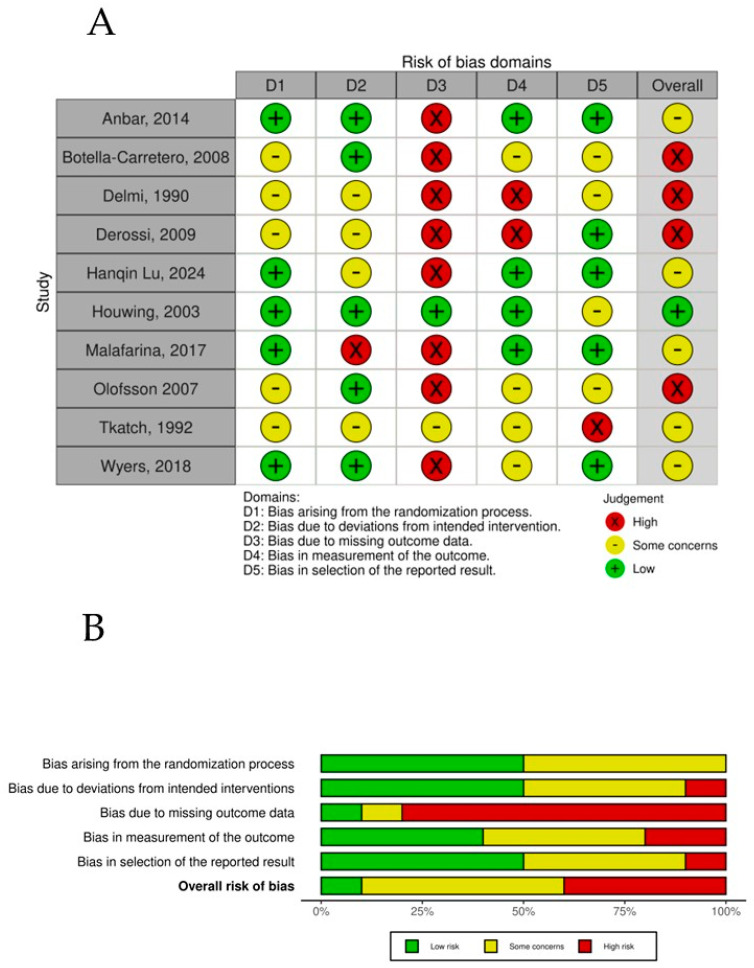
(**A**) Risk of bias per study. (**B**) Risk-of-bias summary plot.

The overall risk-of-bias assessment across the randomized controlled trials highlights key trends and recurring challenges within the evaluated dimensions (Figure 7). Bias arising from the randomization process (D1) showed a balanced distribution, with 50% of studies rated as low risk and the remaining 50% presenting some concerns, showing that RCTs might have limitations in controlling for potential confounders, which could influence the validity of the reported results in those studies. Owing to deviations from the intended intervention (D2) dimension, the majority of studies (60%) achieved a low-risk rating, indicating, in general, appropriate and systematic selection processes. Some concerns were observed in 30% of the studies, reflecting issues such as unclear inclusion criteria or potential selection bias. Bias due to missing outcome data (D3) emerged as the most problematic domain, with 80% of studies classified as high risk. This finding underscores consistent difficulties in ensuring accurate blindness of the participants to the intervention in the trials. Most of the trials recognized that, owing to the characteristics of the intervention, it was impossible to blind it, but most of the trials also failed to blind the assessors to the intervention, which in general could always be possible. Bias in the measurement of the outcome (D4) showed greater variability, with 50% at low risk, 30% at high risk, and 20% at high risk, reflecting potential deviations from planned interventions that may have influenced the outcomes and introduced potential bias. Finally, bias in the selection of the reported results (D5) was relatively favorable, with 50% of the studies classified as low risk, suggesting minimal data loss and adequate handling of incomplete information. In contrast, some concerns were noted in 40% of the studies, indicating challenges with missing data that could affect the robustness of the findings. A small proportion (10%) displayed high risk in this domain. When the overall risk of bias was evaluated, a pattern of moderate concern emerged. Fifty percent of the studies were rated as presenting some concerns, indicating a moderate level of bias that reflects a combination of minor to moderate limitations across dimensions. Importantly, 40% of the studies were classified as high risk, driven predominantly by issues in intervention classification (D3) and deviations from intended interventions (D4).

**Figure 8:**
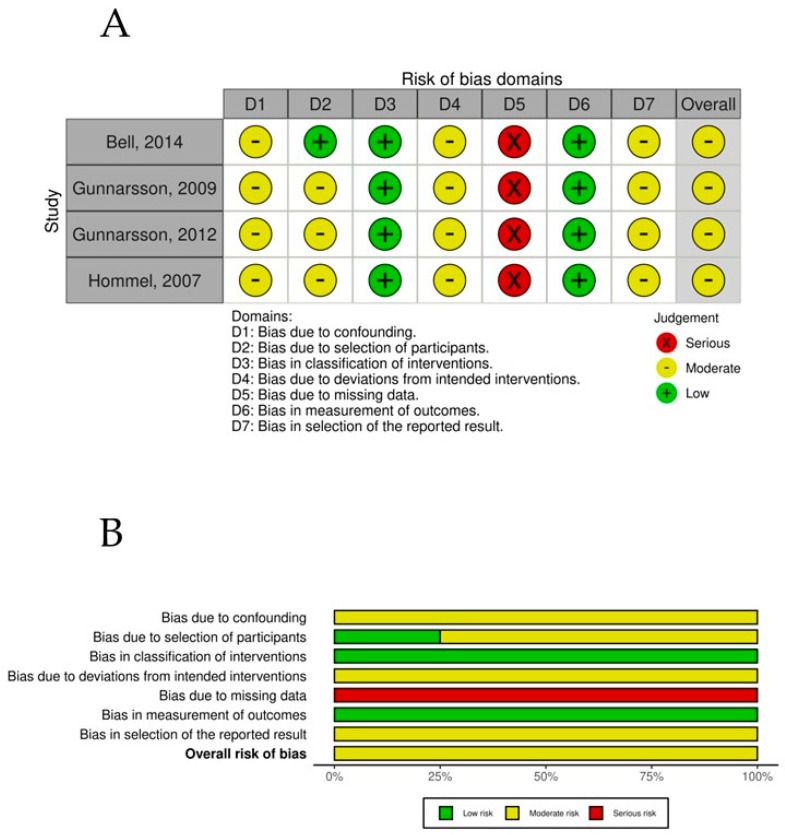
(**A**) ROBINS-1 per study. (**B**) ROBINS-1 summary plot.

The overall risk-of-bias assessment for the nonrandomized controlled trials was assessed using the ROBINS-I tool. All studies demonstrated a moderate overall risk of bias, driven primarily by recurring issues in confounding (D1) and missing data (D5). Specifically, confounding bias (D1) was classified as moderate in all studies, reflecting the inherent limitations of nonrandomized designs, a common methodological challenge that could influence the observed outcomes. The missing data domain (D5) posed the most significant risk, with all studies being rated as serious (derived from high levels of incomplete data often caused by early participant dropouts or incomplete follow-up), affecting the reliability and completeness of the reported results. In contrast, the classification of interventions (D3) and the measurement of outcomes (D6) demonstrated strength across all studies, with a consistent rating of low risk of bias. Moderate risks were also observed in deviations from intended interventions (D4) and selection of reported results (D7), which were recurrent across all studies (Figure 8).

### 3.6. Publication Bias

**Figure 9:**
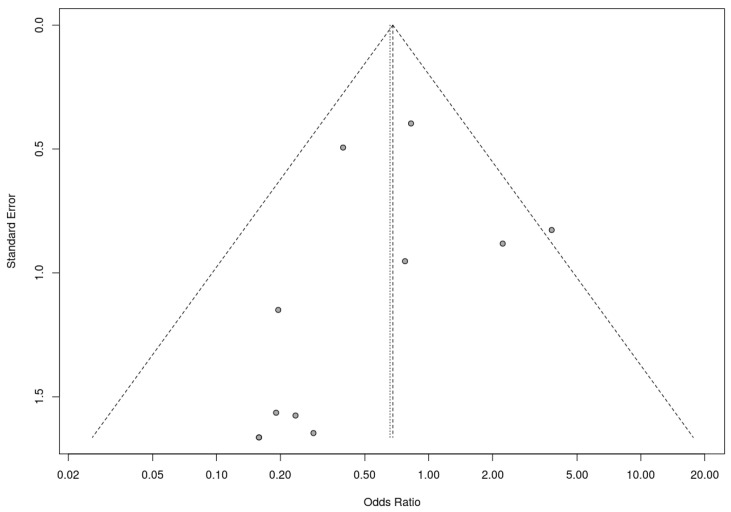
Funnel plot for randomized controlled trials included in the meta-analysis.

A trim-and-fill analysis was used to assess the presence of risk of bias. In this analysis, RCTs and nonrandomized studies were analyzed separately, as the presence of publication bias among randomized controlled trials was assessed by trim-and-fill analysis. This analysis suggested the addition of two studies to address funnel plot asymmetry (Figure 9). This led to a slightly adjusted overall effect size according to the random effects model. Fail-safe N analysis using the Rosenthal approach indicated the need for 10 studies with null results to override the observed significance level. Overall, the results obtained suggest an absence of risk of publication bias.

**Figure 10:**
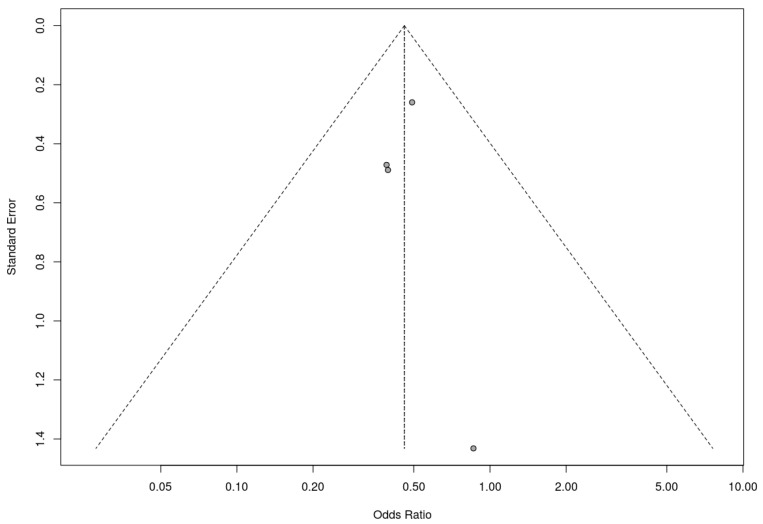
Funnel plot for nonrandomized controlled trials included in the meta-analysis.

Similarly, a trim-and-fill analysis was used for the included nonrandomized controlled trials, which did not add any studies, suggesting no evidence of publication bias. The fail-safe N calculation result was 3 (three additional studies with null results would be needed for the observed significance level to exceed the conventional threshold of 0.05). In this case, these results must be assessed with caution due to the small number of studies included in the analysis. Nevertheless, the results obtained suggest that the results of the meta-analysis are robust to the possibility of publication bias. The associated funnel plot is shown in Figure 10.

## 4. Discussion

In this study, we conducted a comprehensive systematic review and meta-analysis of randomized and nonrandomized controlled trials that investigated the effects of oral nutritional interventions in patients with hip fractures. This study focused on studies that evaluated the incidence of pressure ulcers as either a primary or secondary outcome. Individual analysis of the 14 studies included in the meta-analysis indicated that only 2 studies showed statistically significant results regarding the beneficial effect of oral nutritional intervention on the risk of pressure ulcer development, whereas 12 studies did not show statistical significance for their interventions. However, the pooled analysis revealed an overall favorable effect for this type of intervention. The findings reported here suggest that although the individual studies may lack sufficient statistical power or show variability in their results, the pooled analysis supports the efficacy of oral dietary interventions in these patients and that, therefore, oral nutritional supplementation in patients recovering from hip fractures may have a significant effect on reducing the risk of pressure ulcer development.

In hip fracture care, various nutritional strategies have been explored to improve recovery and reduce complications. These interventions vary in their approach, ranging from comprehensive, multidisciplinary programs to the specific use of nutritional supplements, to precise adjustments in caloric intake and nutritional support during the perioperative period [21,22,24,25]. One prominent model is multidisciplinary nutrition care, which considers nutrition as a fundamental pillar of treatment. This type of intervention emphasizes coordination between different professionals as a key element in improving the nutritional status of patients [22]. In-parallel, diversified nursing interventions take a holistic perspective by combining psychological support with rehabilitation education, exercise plans, and personalized dietary adjustments [33]. In terms of nutritional supplementation, a common strategy is the use of ONSs. These supplements can include protein powder to increase protein intake, energy and protein formulas to provide additional calories, and multicomponent supplements that combine L-carnitine, calcium, magnesium, vitamin D3, and L-leucine to improve bone and muscle metabolism [21,25]. In addition to ONSs, hyperproteic diets are also used to accelerate functional recovery, often by adding protein solutions to the usual diet that are sometimes supplemented with calcium and vitamin D [25,28]. Another aspect of intervention focuses on the specific caloric needs of each patient. In this regard, nutritional intervention adjusts caloric intake through oral supplements [23]. Nutritional support also extends to the perioperative period. In these studies, perioperative nutritional intervention combines pre-operative carbohydrate drinks, intravenous glucose infusion, and drinkable nutritional supplements after surgery [25,27]. Finally, multifactorial interventions combine nutrition with other care strategies, such as early rehabilitation, physical therapy, and pressure ulcer risk assessment [5,31].

Some studies have shown that although no significant differences in pressure ulcer incidence between intervention and control groups could be identified, differences in ulcer severity could be detected [26]. In this meta-analysis, we were able to assess the influence of oral dietary intervention on pressure ulcer severity using a subgroup analysis, although most studies did not report a detailed analysis of ulcer classification. This highlights a limitation in interpreting the available results, with most studies reflecting data on the incidence but not the severity of ulcers. The results showed that the intervention did not significantly influence the incidence of stage I pressure ulcers; in contrast, it substantially affected the incidence of stage II ulcers, suggesting that nutritional intervention in hip fracture patients could reduce their incidence. This finding is consistent with previous results suggesting that advanced-stage pressure ulcers may respond better to nutritional intervention. This effect may be due to their greater dependence on systemic factors, such as protein and energy balance, which are critical for wound healing [32]. Thus, oral nutritional supplementation may play a role in delaying the progression of existing pressure ulcers to more advanced and severe stages rather than preventing their initial development. As discussed later, this may also be closely related to the timing of initiation of supplementation, as in many cases, the pressure ulcer will already be in its early stages when supplementation begins. The findings of Houwing et al. [26] are consistent with those of Delmi et al. [24], who reported no significant effect of one month of nutritional supplementation in the immediate post-operative period. However, Delmi et al. reported that benefits became evident during the recovery phase and were observed up to six months after surgery. The potential of nutritional interventions to influence the evolution and severity of pressure ulcers over time, particularly during convalescence periods, is therefore highlighted. Continued supplementation after hospital discharge therefore also appears to be important, especially when combined with a rehabilitation program, with patients receiving post-discharge supplementation being observed to have a continued improvement in nutritional status and a lower incidence of long-term complications [24,25]. Unfortunately, owing to the limited data on ulcer severity in most studies included in this meta-analysis, we were only able to partially evaluate this aspect. The ideal duration of oral supplementation to reduce the risk of pressure ulcers in hip fracture patients appears to be a combination of timely intervention, continuous supplementation during hospitalization and possibly also after discharge, and the use of supplements with an appropriate composition. Future research should prioritize the detailed reporting of ulcer severity and progression to better understand the full impact of nutritional interventions on this critical outcome. Although oral supplementation may not completely prevent pressure ulcers, studies suggest that it may be effective in reducing the incidence of grade II ulcers, delaying their progression and shortening their duration. The effectiveness of oral supplementation is greatest when it is started early, at the right dose and in combination with other preventive measures.

The inability to reduce the incidence of pressure ulcers in some studies may also be attributed to the timing of the nutritional intervention, which was initiated after the critical period for its formation, often immediately following surgery, in most of the studies. In fact, evidence from an animal study suggests that pressure ulcers can develop as a result of ischemia–reperfusion injury followed by inflammation, which begins only after the cessation of prolonged, unrelieved pressure, indicating that a window for effective intervention may occur earlier [26,34]. Maximizing the potential benefits that oral nutritional supplementation may have on preventing the risk of pressure ulcer development or its aggravation, which may therefore require intervention to be initiated much earlier, potentially during the preoperative or early recovery phase. We stress the importance of considering the timing of dietary intervention when considering its effects on pressure ulcer prevention and treatment.

In our study, it was not possible to collect sufficient results to evaluate the influence of interventions based on oral nutritional supplements and/or nutritional supplementation via tubes in hip fracture patients. In the literature, we were only able to detect the study by Hartgrink et al. [35], which did not report statistically significant differences in the incidence or severity of pressure ulcers between the two types of interventions. In this context, the study by Houwing et al., which was included in the analysis, reported tube feeding in some hip fracture patients and revealed no statistically significant differences. However, this study, in absolute terms, reported fewer pressure ulcers in patients who received tube feeding [34]. Despite this trend, it was not possible to analyze sufficient data to include tube feeding as an additional independent variable in the meta-analysis. It is therefore difficult to draw definitive conclusions about the efficacy of oral and tube supplementation in the prevention and treatment of pressure ulcers.

The studies reviewed in this paper, taken together, suggest that specialized nutritional treatment should be a fundamental part of all treatment strategies in patients who have suffered a hip fracture. Health status, as well as isolation or disease-related complications, often causes nutritional deficiencies in these patients, increasing their risk of developing vitamin and amino acid deficits. In these patients who are generally characterized by advanced age, this situation may be aggravated by the presence of comorbidities such as dementia. Given the importance that the nutritional aspect seems to have in the risk of developing pressure ulcers, it is striking that multidisciplinary teams tend to lack nutrition specialists in their composition, representing only 26.0% of the teams, which creates an essential care deficit to obtain optimal results in patients [36].

A series of limitations identified in the studies analyzed should be highlighted. On the one hand, low adherence to treatment with oral supplements is a common problem, which significantly decreases their effectiveness [21,29]. Some patients may find supplements unappetizing or experience side effects such as nausea and diarrhea [21]. There is great variability in study designs, including different patient populations, types of supplements, doses, durations of intervention, and outcome endpoints. This makes it difficult to compare and draw definitive conclusions [29]. The lack of fully adequate control groups and lack of blinding in some studies limit the validity of the study findings [27]. Several studies began supplementation after pressure ulcers developed, which may have limited its effectiveness in primary prevention [26]. Pressure ulcers often develop in the first few days after surgery, suggesting that intervention should be performed earlier, even before surgery [26,30]. Some studies had short intervention periods, which may not have been sufficient to observe a significant effect on pressure ulcer prevention and healing [30]. Across studies, there is no consensus on the optimal composition of supplements for pressure ulcer prevention. Other factors, such as mobility and general health status, may influence the risk of pressure ulcer development, making it difficult to isolate the specific effect of nutritional supplementation [26]. Some studies considered only the incidence of ulcers without assessing the severity and speed of ulcer healing, and the lack of long-term follow-up in many studies limits the understanding of the benefits of supplementation in preventing the risk of ulcer onset, development, and progression [23,30]. In addition, we must recognize that nonrandomized trials have been incorporated in the meta-analysis, although conveniently analyzed by means of subgroups. Together, these studies while maintained methodological rigor in certain domains, such as intervention classification and outcome measurement, persistent issues with confounding, deviations from interventions, and missing data underline the moderate overall risk of bias observed across the studies. These limitations emphasize the need for careful interpretation of the findings and underscore areas for improvement in the design and conduct of future nonrandomized studies.

## 5. Conclusions

The incidence and evolution of pressure ulcers can be improved by oral dietary supplementation in patients who have undergone hip fracture surgery. By addressing the commonly diagnosed nutritional deficiencies in this target population, oral nutritional supplementation improves tissue repair, promotes skin integrity, and ultimately reduces the risk of pressure ulcer formation. Accordingly, we propose that oral nutritional supplementation should be considered an essential component of comprehensive post-hip-fracture care.

## Figures and Tables

**Table 1:** Description of the studies included in the meta-analysis.

Author (Year, Location)	Type of Nutritional Intervention	Study Design	Follow-Up	Age (Years)	Sex (Female)	Sample Size	Intervention Characteristics	ONS	Duration of the Intervention	Primary Outcome	Secondary Outcome
Anbar (2024, Israel)	Oral supplement	RCT	Hospital stay	IG: 82.3 ± 6.1 CG: 83.7 ± 6.4	IG: 72.7% CG: 60.7%	IG: 22 CG: 28	Personalized Nutritional Support: Tailored interventions based on individual energy requirements. REE Measurement: Repeated assessments using indirect calorimetry. Dietary Provision: Hospital-prepared diets complemented with oral nutritional supplements. Education: Comprehensive guidance provided to both patients and caregivers.	Ensure Plus and Glucerna (Abbott Laboratories): Ensure Plus (355 kcal, 13.5 g of protein per 237 mL); Glucerna (237 kcal and 9.9 g of protein)	14 days or until hospital discharge, whichever came first	Post-operative complications and length of hospital stay	Daily energy intake and calculated cumulative energy balance during the study period [20]
Bell (2014, Australia)	Oral supplement/tube feeding for patients with inadequate oral intake	Controlled trial	Hospital stay	INC: 81.7 (95% CI: 78.4 to 85.0) MMNC: 82.9 years (95% CI: 80.5 to 85.2)	INC: 72.7% MMNC: 65.8%	INC: 44 MMNC: 38	Multidisciplinary Approach: Involves professionals from various fields. Multimodal Nutritional Care: Utilizes several nutritional strategies.	β-hydroxy-β-methyl butyrate, calcium, and vitamin D. Macronutrient composition: 24% protein, 29% fat, and 46% carbohydrates.	The MMNC model was implemented over a 4-week period. Following this initial period, patients were enrolled under the new care model from July to September 2012 and were monitored until discharge.	Mean 24-hour energy and protein intake and changes in nutritional status.	Changes in nutritional status from admission to discharge, discharge destination, length of hospital stay, incidence of complications (pressure ulcers, surgical wound infections, and aspiration pneumonia), and inpatient mortality. [19]
Botella-Carretero (2008, Spain)	Oral supplement	RCT	Hospital discharge	PPS: 83.1 ± 6.3 EPS: 84.6 ± 5.7 CG: 83.7 ± 7.9	CG: 76.7% PPS: 90% EPS: 70%	PPS: 30 EPS: 30 CG: 30	Protein Powder Supplementation and Energy Protein Supplementation:	Protein powder supplement (Vegenat-med Proteina, Vegenat SA): 9 g of protein and 38 kcal per 10 g packet dissolved in water, milk, or soup. Energy–Protein Supplement (Hyperproteic Resource, Novartis Medical Nutrition): 18.8 g of protein and 250 kcal per 200 mL.	Began 48 h after surgery, was maintained throughout the hospital stay, and continued after discharge	Nutritional status of patients at hospital discharge	Tolerance to oral nutritional supplements, length of hospital stay, post-operative complication, time to mobilization
Delmi (1990, Switzerland)	Oral supplement	RCT	6 months	IG: 80.4 (61–93) CG: 82.9 (66–96)	IG: 88.9% CG: 90.6%	IG: 27 CG: 32	Oral Nutritional Supplements: High-energy, high-protein content	250 mL oral formula providing 254 kcal and 20.4 g of protein per serving. It contained 29 g of carbohydrates, 5.8 g of lipids, and 525 mg of calcium, along with essential vitamins (A, D3, E, B1, B2, B6, B12, C, nicotinamide, folate, calcium pantothenate, and biotin) and minerals.	Mean: 32 days	Clinical course during hospitalization and at 6 months assessed by frequency of complications, mortality rates, overall clinical outcomes	Nutritional status improvement; length of hospital stay; energy, protein, and calcium intake; incidence of pressure ulcers; functional recovery
Derossi (2009, Italy)	Oral supplement	RCT	40–50 days after hospitalization	IG: 79.9 ± 7.3 CG: 80.4 ± 6.8	IG: 83.3 CG: 84.9	IG: 54 CG: 53	Daily nutritional supplement (Restorfast^TM^)	Restorfast™: L-carnitine (345 mg), calcium (500 mg), magnesium (250 mg), vitamin D_3_ (5 µg), and L-leucine (500 mg).	6 weeks	Functional recovery of patients assessed through a > 50% improvement in the Barthel Index Instrumental Activities of Daily Living score combined BI and IADL scores compared to prefracture levels at discharge and at the end of the study	Length of hospital stay, body mass index, brachial circumference, complication rates, plasma albumin, and hemoglobin
Houwing (2003, Netherlands)	Oral supplement	RCT	28 days or until hospital discharge	IG: 81.5 ± 0.9 CG: 80.5 ± 1.3	IG: 78.4% CG: 84.6%	IG: 51 CG: 52	High-protein supplement enriched with arginine, zinc, and antioxidants.	Each 100 mL provided 125 kcal, 10 g of protein, 1.5 g of L-arginine, 5 mg of zinc, 125 mg of vitamin C, 50 mg of vitamin E, and 1 mg of carotenoids.	4 weeks	Incidence of pressure ulcers	Stage of pressure ulcers, time of onset, number of days with prevalent pressure ulcers, total maximum wound size
Malafarina (2017, Spain)	Oral supplement	RCT	Mean: 42.3 ± 20.9 days	IG: 85.7 ± 6.5 CG: 84.7 ± 6.3	IG: 63.7% CG: 81.4%	IG: 49 CG: 52	Dietary Regimen: Standard diet supplemented with oral nutritional supplementation.	Ensure^®^ Plus Advance (Abbott Laboratories). Each 220 mL bottle provided 660 kcal/day. Macronutrient composition: 24% protein, 29% fat, and 46% carbohydrates. The supplement was also enriched with β-hydroxy-β-methylbutyrate (0.7 g/100 mL), calcium (227 mg/100 mL), and vitamin D (25(OH)D, 227 IU/100 mL).	Mean: 42.3 ± 20.9 days	Change in appendicular lean mass	Nutritional and biochemical markers: Changes in body mass index, protein concentration (total protein, albumin, transthyretin), and vitamin D (25(OH)D) levels. Functional outcomes: Barthel Index and the Functional Ambulation Categories (FACs) score. Inflammatory markers: Changes in C-reactive protein (CRP), interleukin-1 (IL-1), interleukin-6 (IL-6), and tumor necrosis factor-alpha (TNF-α) levels. Muscle function and strength: Handgrip strength and Grip Work Index (GWI). Gait speed at discharge.
Olofsson (2007, Sweden)	Oral supplement	RCT	4 months	IG: 82.1 ± 6.8 CG: 82.2 ± 5.6	IG: 75% CG: 77%	IG: 83 CG: 74	Multifactorial Nutritional Program: Nutritional journal, protein-enriched meals, nutritional and protein drinks, nutritional support, rehabilitation support, customized meal environment	Protein-enriched meals and oral nutritional supplementation. Patients received at least two high-protein and high-energy drinks per day throughout hospitalization, along with individualized dietary adjustments	At least four days post-operatively, with the nutritional support continuing throughout the entire hospitalization period	Delirium and pressure ulcers	Nutritional parameters, length of hospitalization, other post-operative complication, and cognitive and mental health status
Tkatch (1992, Switzerland)	Oral supplement	RCT	7 months	IG: 83.2 ± 1.3 CG: 81.3 ± 1.6	IG: 90.9% CG: 82.8%	IG: 33 CG: 29	Oral Nutritional Supplement: Contained protein, vitamins, and minerals. Control Group: Received a similar supplement without protein.	20.4 g of protein (from milk), 5.8 g of lipids, and 29.5 g of carbohydrates. Fortified with calcium (0.525 g), magnesium (70 mg), phosphorus (270 mg), vitamin A (750 IU), and vitamin D3 (25 IU)	Approximately 38 days	Rate of complications, mortality during the hospital stay and 7 months after the fracture, and length of hospital stay	Bone mineral density, plasma osteocalcin level, nutritional status, and fracture incidence
Wyers (2018, Netherlands)	Oral supplement	RCT	5 años	IG: 77 SEM (1.) CG: 76 SEM (1.1)	IG: 74.0 CG: 68.4%	IG: 73 CG: 79	Dietetic counseling, a high-protein and high-energy diet, and daily oral nutritional supplementation	Cubitan (N.V. Nutricia, Zoetermeer, Netherlands). Each 400 mL daily dose provided 500 kcal and 40 g of protein	Three months post-surgery	Total length of stay	Nutritional status: Energy and nutrient intake, body weight, BMI, mid-upper arm circumference, skinfold thickness, and handgrip strength. Functional outcomes: Mobility, independence, and quality of life. Cognitive function and psychological well-being. Post-operative complications: Infections, cardiovascular events, pressure ulcers, delirium, and anemia. Subsequent fractures and mortality: Followed at one and five years.
Gunnarson (2009, Sweden)	Oral supplement	Controlled Trial	5 days	IG: 81.5 ± 9.0 CG: 80.9 ± 8.4	IG: 66% CG: 76%	IG: 50 CG: 50	Pre-operative Nutrition: Glucose infusion. Four carbohydrate supplement drinks. Post-operative Nutrition (for five days): Nutritional supplement drinks administered three times daily.		5 days	Incidence of pressure ulcers	Nutrient and liquid intake, weight changes, nosocomial infections, cognitive ability, walking assistance needs, functional ability, and length of hospital stay.
Gunnarson (2012, Sweden)	Oral supplement	Controlled trial	5 days	IG: 81.3 ± 9 CG: 80.8 ± 8.1	IG: 67.4% CG:73.8%	IG:46 CG:42	Pre-operative phase: Carbohydrate drinks and intravenous glucose infusion. Post-operative phase (five days): Nutritional supplements	Carbohydrate supplementation: 100 kcal per 200 mL along with an intravenous glucose infusion (50 mg/mL). Post-operative phase: nutritional supplement (300 kcal per 200 mL, for five days)	5 days	Nutritional biochemical markers (S-IGF-1, S-transthyretin, and S-albumin)	Post-operative complications: Incidence of hospital-acquired pressure ulcers and incidence of hospital-acquired infections. Energy intake: Comparison of calorie intake between groups. Length of hospital stay.
Hanqin Lu (2024, China)	Oral supplement	RCT	Hospital discharge	IG: 71.13 ± 7.26 CG: 71.16 ± 7.30	IG: 44.2% CG: 46.5%	IG: 43 CG: 43	Nutritional support: Oral enteral nutrition powder (Ansul), dietary recommendations, and diversified nursing measures	Ansul enteral nutrition powder per 100 g: 450 kcal and 15.9 g of protein. Main ingredients: corn starch, casein sodium, casein calcium, minerals, vitamins, and trace elements	7 days	Nutritional status and the incidence of post-operative complications (e.g., pressure ulcers)	Functional recovery, pain levels, emotional well-being, activities of daily living, complication rates, time to mobilization, hospital length of stay, and patient satisfaction
Hommel (2017, Sweden)	Oral supplement	Controlled trial	Hospital stay + 4 months + 12 months	IG: 81.5 ± 10.5 CG: 80.4 ± 10.3	IG: 70.5% CG: 66.7%	IG: 210 CG: 210	Post-operative nutrition: Patients were given an oral milk-based nutritional supplement twice daily.	Milk-based drink: each 100 mL provided 125 kcal. Enriched with arginine, zinc, vitamins A, B, C, and E, as well as antioxidants (selenium and carotenoids)	10.8 days for the control group and 11.8 days for the intervention group.	Incidence of pressure ulcers	Nutritional status, anthropometric measurements, pressure ulcer severity, complication rates, functional outcomes, and long-term recovery

IG: intervention; CG: control group: PPS: protein powder supplement; EPS: energy–protein supplement; MMNC: multimodal nutritional care; INC: individualized nutritional care.

## Data Availability

All data used in this meta-analysis can be retrieved from the original sources by accessing the databases specified in the methodology section. The study is fully reproducible in this regard.

## Supplementary Materials

The following supporting information can be downloaded at https://www.mdpi.com/article/10.3390/nu17040644/s1: Supplementary File S1. PRISMA 2020 checklist [13]; Supplementary File S2. Search strategies.
