# Supplementary material for: Nutritional Interventions for Pressure Ulcer Prevention in Hip Fracture Patients: A Systematic Review and Meta-Analysis of Controlled Trials
Source: Nutrients. 2025 Feb 11;17(4):644. doi: 10.3390/nu17040644 (PMC11858231; doi:10.3390/nu17040644)
Supplement: Supplementary file 1 [file nutrients-17-00644-s001.zip › Search Strategy.pdf]

PUBMED

("fractur"[All Fields] OR "fractural"[All Fields] OR "fracture s"[All Fields] OR "fractures, bone"[MeSH Terms] OR ("fractures"[All Fields] AND "bone"[All Fields]) OR "bone fractures"[All Fields] OR "fracture"[All Fields] OR "fractured"[All Fields] OR "fractures"[All Fields] OR "fracturing"[All Fields] OR ("fractur"[All Fields] OR "fractural"[All Fields] OR "fracture s"[All Fields] OR "fractures, bone"[MeSH Terms] OR ("fractures"[All Fields] AND "bone"[All Fields]) OR "bone fractures"[All Fields] OR "fracture"[All Fields] OR "fractured"[All Fields] OR "fractures"[All Fields] OR "fracturing"[All Fields])) AND ("hip"[MeSH Terms] OR "hip"[All Fields] OR ("pelvi"[All Fields] OR "pelvis"[MeSH Terms] OR "pelvis"[All Fields]) OR ("femur"[MeSH Terms] OR "femur"[All Fields] OR "femurs"[All Fields] OR "femur s"[All Fields] OR "femural"[All Fields] OR "femure"[All Fields]) OR ("femor"[All Fields] OR "femorals"[All Fields] OR "femur"[MeSH Terms] OR "femur"[All Fields] OR "femoral"[All Fields])) AND ((("dietary supplements"[MeSH Terms] OR ("dietary"[All Fields] AND "supplements"[All Fields]) OR "dietary supplements"[All Fields] OR "supplement"[All Fields] OR "supplement s"[All Fields] OR "supplemented"[All Fields] OR "supplementing"[All Fields] OR "supplements"[All Fields] OR ("supplemental"[All Fields] OR "supplementating"[All Fields] OR "supplementation"[All Fields] OR "supplementation s"[All Fields] OR "supplementations"[All Fields] OR "supplemention"[All Fields]) OR ("dietary supplements"[MeSH Terms] OR ("dietary"[All Fields] AND "supplements"[All Fields]) OR "dietary supplements"[All Fields] OR "supplement"[All Fields] OR "supplement s"[All Fields] OR "supplemented"[All Fields] OR "supplementing"[All Fields] OR "supplements"[All Fields])) AND ("nutrition s"[All Fields] OR "nutritional status"[MeSH Terms] OR ("nutritional"[All Fields] AND "status"[All Fields]) OR "nutritional status"[All Fields] OR "nutrition"[All Fields] OR "nutritional sciences"[MeSH Terms] OR ("nutritional"[All Fields] AND "sciences"[All Fields]) OR "nutritional sciences"[All Fields] OR "nutritional"[All Fields] OR "nutritionals"[All Fields] OR "nutritions"[All Fields] OR "nutritive"[All Fields] OR ("nutrition s"[All Fields] OR "nutritional status"[MeSH Terms] OR ("nutritional"[All Fields] AND "status"[All Fields]) OR "nutritional status"[All Fields] OR "nutrition"[All Fields] OR "nutritional sciences"[MeSH Terms] OR ("nutritional"[All Fields] AND "sciences"[All Fields]) OR "nutritional sciences"[All Fields] OR "nutritional"[All Fields] OR "nutritionals"[All Fields] OR "nutritions"[All Fields] OR "nutritive"[All Fields]) OR ("nutrient s"[All Fields] OR "nutrients"[MeSH Terms] OR "nutrients"[All Fields] OR "nutrient"[All Fields]))) OR ("nutrition s"[All Fields] OR "nutritional status"[MeSH Terms] OR ("nutritional"[All Fields] AND "status"[All Fields]) OR "nutritional status"[All Fields] OR "nutrition"[All Fields] OR "nutritional sciences"[MeSH Terms] OR ("nutritional"[All Fields] AND "sciences"[All Fields]) OR "nutritional sciences"[All Fields] OR "nutritional"[All Fields] OR "nutritionals"[All Fields] OR "nutritions"[All Fields] OR "nutritive"[All Fields])) AND ((("patient s"[All Fields] OR "patients"[MeSH Terms] OR "patients"[All Fields] OR "patient"[All Fields] OR "patients s"[All Fields]) AND "s"[All Fields]) OR ("patient s"[All

Fields] OR "patients"[MeSH Terms] OR "patients"[All Fields] OR "patient"[All Fields] OR "patients s"[All Fields])) Filters: from 1000/1/1 - 2024/4/30

SCOPUS

(ALL (fractur) OR ALL (fractural) OR ALL ("fracture s") OR INDEXTERMS ("fractures, bone") OR (ALL (fractures) AND ALL (bone)) OR ALL ("bone fractures") OR ALL (fracture) OR ALL (fractured) OR ALL (fractures) OR ALL (fracturing) OR (ALL (fractur) OR ALL (fractural) OR ALL ("fracture s") OR INDEXTERMS ("fractures, bone") OR (ALL (fractures) AND ALL (bone)) OR ALL ("bone fractures") OR ALL (fracture) OR ALL (fractured) OR ALL (fractures) OR ALL (fracturing))) AND (INDEXTERMS (hip) OR ALL (hip) OR (ALL (pelvi) OR INDEXTERMS (pelvis) OR ALL (pelvis)) OR (INDEXTERMS (femur) OR ALL (femur) OR ALL (femurs) OR ALL ("femur s") OR ALL (femural) OR ALL (femure)) OR (ALL (femor) OR ALL (femorals) OR INDEXTERMS (femur) OR ALL (femur) OR ALL (femoral))) AND (((INDEXTERMS ("dietary supplements") OR (ALL (dietary) AND ALL (supplements)) OR ALL ("dietary supplements") OR ALL (supplement) OR ALL ("supplement s") OR ALL (supplemented) OR ALL (supplementing) OR ALL (supplements) OR (ALL (supplemental) OR ALL (supplementating) OR ALL (supplementation) OR ALL ("supplementation s") OR ALL (supplementations) OR ALL (supplemention)) OR (INDEXTERMS ("dietary supplements") OR (ALL (dietary) AND ALL (supplements)) OR ALL ("dietary supplements") OR ALL (supplement) OR ALL ("supplement s") OR ALL (supplemented) OR ALL (supplementing) OR ALL (supplements))) AND (ALL ("nutrition s") OR INDEXTERMS ("nutritional status") OR (ALL (nutritional) AND ALL (status)) OR ALL ("nutritional status") OR ALL (nutrition) OR INDEXTERMS ("nutritional sciences") OR (ALL (nutritional) AND ALL (sciences)) OR ALL ("nutritional sciences") OR ALL (nutritional) OR ALL (nutritionals) OR ALL (nutritions) OR ALL (nutritive) OR (ALL ("nutrition s") OR INDEXTERMS ("nutritional status") OR (ALL (nutritional) AND ALL (status)) OR ALL ("nutritional status") OR ALL (nutrition) OR INDEXTERMS ("nutritional sciences") OR (ALL (nutritional) AND ALL (sciences)) OR ALL ("nutritional sciences") OR ALL (nutritional) OR ALL (nutritionals) OR ALL (nutritions) OR ALL (nutritive)) OR (ALL ("nutrient s") OR INDEXTERMS (nutrients) OR ALL (nutrients) OR ALL (nutrient)))) OR (ALL ("nutrition s") OR INDEXTERMS ("nutritional status") OR (ALL (nutritional) AND ALL (status)) OR ALL ("nutritional status") OR ALL (nutrition) OR INDEXTERMS ("nutritional sciences") OR (ALL (nutritional) AND ALL (sciences)) OR ALL ("nutritional sciences") OR ALL (nutritional) OR ALL (nutritionals) OR ALL (nutritions) OR ALL (nutritive))) AND (((ALL ("patient s") OR INDEXTERMS (patients) OR ALL (patients) OR ALL (patient) OR ALL ("patients s")) AND ALL (s)) OR (ALL ("patient s") OR INDEXTERMS (patients) OR ALL (patients) OR ALL (patient) OR ALL ("patients s"))) AND (

EXCLUDE (SRCTYPE , "b") OR EXCLUDE (SRCTYPE , "k") OR EXCLUDE (SRCTYPE , "p")) AND (EXCLUDE (DOCTYPE , "re") OR EXCLUDE (DOCTYPE , "cp") OR EXCLUDE (DOCTYPE , "ed") OR EXCLUDE (DOCTYPE , "le") OR EXCLUDE (DOCTYPE , "tb") OR EXCLUDE (DOCTYPE , "no")) AND (LIMIT-TO (EXACTKEYWORD , "Randomized Controlled Trial"))

WOS

(fracture OR fractural OR "fracture s" OR "fractures, bone" OR (fractures AND bone) OR "bone fractures" OR fracture OR fractured OR fractures OR fracturing OR (fracture OR fractural OR "fracture s" OR "fractures, bone" OR (fractures AND bone) OR "bone fractures" OR fracture OR fractured OR fractures OR fracturing)) AND (hip OR hip OR (pelvic OR pelvis OR pelvis) OR (femur OR femur OR femurs OR "femur s" OR femoral OR femurs) OR (femur OR femoralis OR femur OR femur OR femoral)) AND ((("dietary supplements" OR (dietary AND supplements) OR "dietary supplements" OR supplement OR "supplement s" OR supplemented OR supplementing OR supplements OR (supplemental OR supplementaling OR supplementation OR "supplementation s" OR supplementation OR supplementaion) OR ("dietary supplements" OR (dietary AND supplements) OR "dietary supplements" OR supplement OR "supplement s" OR supplemented OR supplementing OR supplements)) AND ("nutrition s" OR "nutritional status" OR (nutritional AND status) OR "nutritional status" OR nutrition OR "nutritional sciences" OR (nutritional AND sciences) OR "nutritional sciences" OR nutritional OR nutritionals OR nutritious OR nutritive OR ("nutrition s" OR "nutritional status" OR (nutritional AND status) OR "nutritional status" OR nutrition OR "nutritional sciences" OR (nutritional AND sciences) OR "nutritional sciences" OR nutritional OR nutritionals OR nutritious OR nutritive) OR ("nutrient s" OR nutrients OR nutrients OR nutrient))) OR ("nutrition s" OR "nutritional status" OR (nutritional AND status) OR "nutritional status" OR nutrition OR "nutritional sciences" OR (nutritional AND sciences) OR "nutritional sciences" OR nutritional OR nutritionals OR nutritious OR nutritive)) AND ((("patient s" OR patients OR patients OR patient OR "patients s") AND s) OR ("patient s" OR patients OR patients OR patient OR "patients s")) (Topic) and Review Article or Meeting or Case Report or Awarded Grant or Letter or Editorial Material or Retracted Publication or Book or Patent or Publication With Expression Of Concern or Biography (Exclude – Document Types)

OVID MEDLINE

(fractur.af. OR fractural.af. OR "fracture s".af. OR exp "fractures, bone"/ OR (fractures.af. AND bone.af.) OR "bone fractures".af. OR fracture.af. OR fractured.af. OR fractures.af. OR

fracturing.af. OR (fractur.af. OR fractural.af. OR "fracture s".af. OR exp "fractures, bone"/ OR (fractures.af. AND bone.af.) OR "bone fractures".af. OR fracture.af. OR fractured.af. OR fractures.af. OR fracturing.af.)) AND (exp hip/ OR hip.af. OR (pelvi.af. OR exp pelvis/ OR pelvis.af.) OR (exp femur/ OR femur.af. OR femurs.af. OR "femur s".af. OR femural.af. OR femure.af.) OR (femor.af. OR femorals.af. OR exp femur/ OR femur.af. OR femoral.af.)) AND (((exp "dietary supplements"/ OR (dietary.af. AND supplements.af.) OR "dietary supplements".af. OR supplement.af. OR "supplement s".af. OR supplemented.af. OR supplementing.af. OR supplements.af. OR (supplemental.af. OR supplementating.af. OR supplementation.af. OR "supplementation s".af. OR supplementations.af. OR supplemention.af.) OR (exp "dietary supplements"/ OR (dietary.af. AND supplements.af.) OR "dietary supplements".af. OR supplement.af. OR "supplement s".af. OR supplemented.af. OR supplementing.af. OR supplements.af.)) AND ("nutrition s".af. OR exp "nutritional status"/ OR (nutritional.af. AND status.af.) OR "nutritional status".af. OR nutrition.af. OR exp "nutritional sciences"/ OR (nutritional.af. AND sciences.af.) OR "nutritional sciences".af. OR nutritional.af. OR nutritionals.af. OR nutritions.af. OR nutritive.af. OR ("nutrition s".af. OR exp "nutritional status"/ OR (nutritional.af. AND status.af.) OR "nutritional status".af. OR nutrition.af. OR exp "nutritional sciences"/ OR (nutritional.af. AND sciences.af.) OR "nutritional sciences".af. OR nutritional.af. OR nutritionals.af. OR nutritions.af. OR nutritive.af.) OR ("nutrient s".af. OR exp nutrients/ OR nutrients.af. OR nutrient.af.))) OR ("nutrition s".af. OR exp "nutritional status"/ OR (nutritional.af. AND status.af.) OR "nutritional status".af. OR nutrition.af. OR exp "nutritional sciences"/ OR (nutritional.af. AND sciences.af.) OR "nutritional sciences".af. OR nutritional.af. OR nutritionals.af. OR nutritions.af. OR nutritive.af.)) AND ((("patient s".af. OR exp patients/ OR patients.af. OR patient.af. OR "patients s".af.) AND s.af.) OR ("patient s".af. OR exp patients/ OR patients.af. OR patient.af. OR "patients s".af.))

EMBASE

(fractur OR fractural OR 'fracture s' OR 'fractures, bone'/exp OR (fractures AND bone) OR 'bone fractures' OR fracture OR fractured OR fractures OR fracturing OR (fractur OR fractural OR 'fracture s' OR 'fractures, bone'/exp OR (fractures AND bone) OR 'bone fractures' OR fracture OR fractured OR fractures OR fracturing)) AND (hip/exp OR hip OR (pelvi OR pelvis/exp OR pelvis) OR (femur/exp OR femur OR femurs OR 'femur s' OR femural OR femure) OR (femor OR femorals OR femur/exp OR femur OR femoral)) AND ((('dietary supplements'/exp OR (dietary AND supplements) OR 'dietary supplements' OR supplement OR 'supplement s' OR supplemented OR supplementing OR supplements OR (supplemental OR supplementating OR supplementation OR 'supplementation s' OR supplementations OR supplemention) OR ('dietary supplements'/exp OR (dietary AND supplements) OR 'dietary supplements' OR supplement OR 'supplement s' OR supplemented OR supplementing OR supplements)) AND ('nutrition s' OR 'nutritional status'/exp OR (nutritional AND status) OR 'nutritional status' OR nutrition OR 'nutritional sciences'/exp OR (nutritional AND sciences) OR 'nutritional sciences' OR nutritional OR nutritionals OR nutritions OR nutritive OR ('nutrition s' OR 'nutritional status'/exp OR (nutritional AND status) OR 'nutritional status' OR nutrition OR 'nutritional sciences'/exp OR (nutritional AND sciences) OR 'nutritional sciences' OR nutritional OR nutritionals OR nutritions OR nutritive) OR ('nutrient s' OR nutrients/exp OR nutrients OR nutrient))) OR ('nutrition s' OR 'nutritional status'/exp OR (nutritional AND

status) OR 'nutritional status' OR nutrition OR 'nutritional sciences'/exp OR (nutritional AND sciences) OR 'nutritional sciences' OR nutritional OR nutritionals OR nutritions OR nutritive)) AND ((('patient s' OR patients/exp OR patients OR patient OR 'patients s') AND s) OR ('patient s' OR patients/exp OR patients OR patient OR 'patients s'))
